# Human DDX56 protein interacts with influenza A virus NS1 protein and stimulates the virus replication

**DOI:** 10.1590/1678-4685-GMB-2020-0158

**Published:** 2021-03-22

**Authors:** Ayşegül Pirinçal, Kadir Turan

**Affiliations:** 1 Marmara University, Institute of Health Sciences, Istanbul, Turkey.; 2 Marmara University, Faculty of Pharmacy, Department of Basic Pharmaceutical Sciences, Istanbul, Turkey.

**Keywords:** Influenza A viruses, NS1 protein, RNA helicase DDX56, virus-host interaction

## Abstract

Influenza A viruses (IAV) are enveloped viruses carrying a single-stranded negative-sense RNA genome. Detection of host proteins having a relationship with IAV and revealing of the role of these proteins in the viral replication are of great importance in keeping IAV infections under control. Consequently, the importance of human DDX56, which is determined to be associated with a viral NS1 with a yeast two-hybrid assay, was investigated for IAV replication. The viral replication in knocked down cells for the DDX56 gene was evaluated. The NS1 was co-precipitated with the DDX56 protein in lysates of cells transiently expressing DDX56 and NS1 or infected with the viruses, showing that NS1 and DDX56 interact in mammalian cells. Viral NS1 showed a tendency to co-localize with DDX56 in the cells, transiently expressing both of these proteins, which supports the IP and two-hybrid assays results. The data obtained with *in silico* predictions supported the *in vitro* protein interaction results. The viral replication was significantly reduced in the DDX56-knockdown cells comparing with that in the control cells. In conclusion, human DDX56 protein interacts with the IAV NS1 protein in both yeast and mammalian cells and has a positive regulatory effect on IAV replication. However, the mechanism of DDX56 on IAV replication requires further elucidation.

## Introduction

Influenza A viruses are enveloped viruses with a single-stranded RNA genome having a negative-polarity. The viral genome consisting of eight RNA segments encodes 11 different proteins (Nakajima, 1997). Non-structural proteins (NS1 and NS2) are synthesized through segment 8, the smallest segment of the viral genome (Lamb and Choppin, 1979). The viral NS1 protein is abundantly synthesized in infected cells, but it is not detected in the virions (Hale *et al*., 2008; Basu *et al*., 2009). The NS2 is synthesized via alternative splicing mechanisms through viral segment 8. The NS1 and NS2 proteins share 10 amino acids in the amino-terminal region. The NS2 protein, which is found in the lowest amount in the virion, has a role in the transport of viral ribonucleoprotein particles (RNPs) with M1 protein and several cellular proteins (Senbas Akyazi *et al*., 2020). Therefore, it is also called a nuclear export protein (NEP) (Neumann *et al*., 2000; Kawaoka and Neumann, 2012). Unlike the NS2 protein, the most important function of NS1 is to cope with the antiviral effects of interferon response mechanisms, which are the intracellular defense system. Therefore, the NS1 protein plays a key role on efficient viral replication in infected cells (Kochs *et al*., 2007). The influenza A virus NS1 protein has an average of 26 kDa molecular weight and consists of 230 or 237 amino acid residues depending on the virus type (Lin *et al*., 2007). This protein has the functional domains for both protein-protein and protein-RNA interactions. The amino-terminal region of the NS1 protein, which contains 1-73 amino acid residues, is the RNA-binding domain and binds to various RNA molecules with low affinity. It has been reported that NS1 protein has a binding ability to the double stranded RNA (dsRNA) molecules formed during viral infection, U6 snRNA, which is a member of the protein/RNA (spliceosome) complex involved in the processing of RNA molecules and the poly-A structure consisting of adenine nucleotides (Hatada and Fukuda, 1992; Qiu *et al*., 1995). The binding of the NS1 protein via the RNA-binding domain to the dsRNA molecules is of great importance in terms of coping with host interferon defense mechanisms (Newby *et al*., 2007). The NS1 protein binds to the dsRNA molecules as a dimeric form (Wang W *et al*., 1999). The amino-terminal region of the NS1 protein is also important for binding to the several cellular proteins. This protein prevents the maturation and transport of cellular mRNAs to the cytoplasm by binding to the cleavage and polyadenylation specificity factor (CPSF) and polyA-binding (PABII) proteins (Nemeroff *et al*., 1998). In contrast, it shows a positive regulatory effect on viral mRNA translation by binding the 5'UTR region of viral mRNAs and PABPI (de la Luna *et al*., 1995; Burgui *et al*., 2003). The region covering the 73-230 amino acid residues of the NS1 protein is the carboxyl-terminal region in the homodimer structure, also known as the effector domain (Xia *et al*., 2009). It has been suggested that the C-terminal part of NS1 has a function in the regulation of IFN α/β, tumor necrosis factor-α (TNF-α) in macrophages infected with influenza A virus, IL-6, and the synthesis of chemokine ligand-3 (CCL3) while the N-terminal domain of the protein inhibits interleukin-1β (IL-1β) and interleukin-18 (IL-18) synthesis (Stasakova *et al*., 2005; Lam *et al*., 2011).

Like other RNA viruses, the influenza viruses use gene expression mechanisms and metabolites of the host cell to replicate by interacting with several cellular proteins. Therefore, the detection of the host cell proteins associated with viral proteins has great importance for the understanding of the viral pathogenesis. In this study, human proteins that may be related to influenza A virus NS1 protein were screened with the yeast two-hybrid method, an effective method for detecting protein interactions, and several candidate proteins previously not reported were identified. The human DDX56 protein, one of the candidate proteins interacting with viral NS1 protein in the yeast cells, was investigated in terms of interaction with NS1 and the effect on the replication of influenza A virus in the mammalian cells. The DDX56 protein, classified into the DEAD-box (Asp-Glu-Ala-Asp) protein family, is an ATP dependent RNA helicase enzyme that plays a role in the RNA metabolism (Zirwes *et al*., 2000). However, it was reported that the DDX56 protein affects the replication of some viruses such as the West Nile virus (WNV) (Xu *et al*., 2011; Xu and Hobman, 2012), FMDV (foot and mouth disease virus) (Fu *et al*., 2019a) and the Sendai virus (SeV) (Fu *et al*., 2019b) by directly interacting with the viral proteins or indirectly with host proteins. In this respect, it may be one of the cellular protein factors that positively regulate infections of influenza A viruses that undergo replication and transcription in the cell nucleus.

## Material and Methods

### Cells, viruses, and antibodies

HEK293 (Human embryonic kidney 293), HeLa (human epithelial carcinoma), and MDCK (Madin-Darby canine kidney) cells were used in transient transfection experiments and/or viral infections. The cells were cultured in Dulbecco's modified Eagle's medium (DMEM) supplemented with 10% (v/v) heat-inactivated fetal calf serum (Gibco, USA), 2 mM L-glutamine, 100 µg/mL streptomycin, 100 IU/mL penicillin, and 1.5 mg/mL sodium bicarbonate at 37 °C in a humidified incubator with 5% CO_2_.

Human influenza virus A/WSN/1933/H1N1 (WSN) and avian influenza virus A/duck/Pennsylvania/10.218/1984/H5N2 (DkPen) were propagated in MDCK cells and/or specific pathogen-free chicken embryos. The viral titer was measured using a standard plaque assay or hemagglutinin (HA) assay (Turan *et al*., 1996). 

Monoclonal mouse anti-HA (Santa Cruz, # sc-7392), polyclonal rabbit anti-NS1 (Invitrogen, # PA5-322439), monoclonal mouse anti-actin (MyBioSource, # MBS9400413), rabbit anti-M1 serum, horseradish peroxidase-conjugated second antibody against species-specific immunoglobulins [anti-mouse IgG-HRP (Invitrogen, # 31420) and/or anti-rabbit IgG-HRP (Invitrogen, # 31423)], Alexa-488-conjugated goat anti-mouse IgG (Abcam, # ab150117) and/or Alexa-568-conjugated goat anti-rabbit IgG (Abcam, # ab175471) antibodies were used in immunoblotting and/or immunofluorescence experiments.

### RNA extraction and first-strand cDNA synthesis

In order for the PCR amplification of human influenza A virus NS1_W_ (WSN) cDNA, the avian influenza A virus NS1_D_ (Dk/Pen) cDNA, DDX56 cDNA and/or quantitation of the related transcripts with revers transcription-quantitative PCR (RT-qPCR), the total RNA was prepared from the cells with the RNeasy plus mini kit (Qiagen, Germany). cDNAs were prepared from 500 ng total RNA derived from HeLa and/or HEK293 cells by using the Moloney murine leukemia virus reverse transcriptase (ReverTraAce, Toyobo Co., Ltd., Japan) and oligo(dT) as a primer for 60 min at 45 °C. The influenza A virus NS1_W_ cDNA and NS1_D_ cDNA were obtained from virus-infected HeLa cells. 

### Constructon of plasmid vectors

All plasmids were constructed using PCR and standard subcloning techniques. The primers used for the PCRs are listed in Table 1.

**Table 1 -:** Oligonucleotide primers used in this study.

Primers name	Sequence (5'-3')
DDX56-For	ATCATGGAGGACTCTGAAGCACTG
DDX56-Rev	ATTCAGGAGGGCTTGGCTGTG
NS1/W-For	TTGAATTCGGAGGATCTGGAATGGATCCAAACACTGTGTC
NS1/W-Rev	TTGAATTCTCAAACTTCTGACCTAATTG
NS1/D-For	TTGAATTCGGAGGATCTGGAATGGACTCCAACACGATAAC
NS1/D-Rev	TTGAATTCTCAAACTTCTGACTCAACTC
DDX56/831-For	CTTGGAACAGTTCAGCATCC
DDX56/959-Rev	AGGACTTCAGCATCAGTTGC
ACTB-For	ATGGAGTCCTGTGGCATCC
ACTB-Rev	CCAGCACAATGAAGATCAAG
AD-Seq-For	AATACCACTACAATGGATGATGT

### Mammalian cell expression plasmids

For the construction of DDX56 expression vectors, the full length of the DDX56 gene consisting of 1644 bp was amplified from HEK293 cDNA with the phosphorylated primer pair DDX56-For and DDX56-Rev. PCR amplification was carried out with a thermostable DNA polymerase (KOD plus, Toyobo Co., Ltd., Japan). The PCR product was purified with an agarose gel extraction kit (QiaexII, Qiagen, Germany). For the construction of the human influenza A virus NS1_W_ and avian influenza A virus NS1_D_ expression vectors, NS1 genes consisting of 693 bp were amplified from virus-infected HeLa cDNA with primer pairs NS1/W-For and NS1/W-Rev or NS1/D-For and NS1/D-Rev. The PCR product was digested with *Eco*RI and purified with an agarose gel extraction kit. 

In order to construct the pCHA-DDX56 plasmid encoding the HA-tagged DDX56 protein (H-DDX56), the DDX56 cDNA was cloned into pCHA (Nagata *et al*., 1998) plasmid digested with *Eco*RV (New England Biolabs, UK) and dephosphorylated with shrimp alkaline phosphatase (Thermo Fisher Scientific, USA). For the pCAGGS-NS1_W_ plasmid vector construction, NS1_W_ cDNA was ligated with pCAGGS (Niwa *et al*., 1991) plasmid digested with *Eco*RI (New England Biolabs, UK) and dephosphorylated with SAP. 

### Yeast cell expression plasmids

In order to construct the pGBD-NS1_W_ and pGBD-NS1_D_ plasmid vectors, the pGBD-C1 (James *et al*., 1996) plasmid was digested with *Eco*RI, dephosphorylated with SAP, and ligated with PCR amplified NS1_W_ cDNA and NS1_D_ cDNA, respectively. For the pACT2-DDX56 plasmid vector construction, the PCR amplified DDX56 cDNA was ligated with pACT2 (Clontech, # 638822) plasmid digested with *Bgl*II, blunted with a Klenow fragment (New England Biolabs, UK) and dephosphorylated with SAP. The nucleotide sequences of all plasmids were confirmed by DNA sequencing.

### Immunoblotting

Immunoblotting analyses were used to detect the expression levels of DDX56 and viral proteins in transiently transfected and/or virus infected cells. Cell lysates were prepared using a RIPA buffer or SDS-PAGE sample buffer. The proteins in lysates were separated using 10% SDS-PAGE and transferred to a polyvinylidene difluoride (PVDF) membrane. After blocking with 5% skim milk, the membrane was incubated with the specific primary antibodies (mouse anti-HA, rabbit anti-NS1, mouse anti-actin and/or rabbit anti-M1 serum) overnight at 4 °C and followed by incubation with a horseradish peroxidase-conjugated second antibody [anti-mouse IgG-HRP and/or anti-rabbit IgG-HRP] for 45 minutes at room temperature. The proteins were visualized with an enhanced chemiluminescence detection kit (GE Healthcare, Italy).

### Yeast two-hybrid screening

The yeast two-hybrid system based on the GAL4 transcription factor was used to identify host proteins that interact with influenza A virus NS1 protein. In brief, the *Saccharomyces cerevisiae* strain PJ69-4A (James *et al*., 1996) [*MAT*a *trp1-901 leu2-3,112 ura3-52 his3-200 gal4(delesyon) gal80(delesyon) LYS2::GAL1-HIS3 GAL2-ADE2 met2::GAL7-lacZ*] was grown in a YPAD medium. The pGBD-NS1_W_ plasmid coding GAL4-BD-NS1_W_ bait fusion protein was transformed into the yeast PJ69-4A strain by using the lithium acetate/polyethylene glycol (LiAc/PEG) and spread on synthetic dropout (SD) agar plates (without Trp) at 30 ^º^C. Then the transformants were selected on a SD(-Trp) plate and checked for the NS1_W_ gene with PCR. The PJ69-4A yeast strain expressing the bait protein was transformed with the large-scale cDNA library derived from HEK293 cells (Clontech, # 638826) and screened following the matchmaker two-hybrid system protocol. Candidates for two hybrid interaction were selected on SD agar plates (without Ade, His, Leu, and Trp) for the primary screening and further tested by the β-galactosidase assay for the second screening. After rescue, the potential positive plasmids carrying cDNAs were isolated using a yeast plasmid DNA miniprep kit (Bio Basic, Canada) according to the manufacturer’s instructions. Competent *E. coli* DH5α were transformed with the plasmids isolated from yeast. Transformants were grown on Luria-Bertani agar plates containing 100 mg/mL ampicillin. Only the clones that were positive for all the reporters and confirmed to be positive by at least two independent tests were selected for specific interactions, sequenced, and identified with BLAST (Basic Local Alignment Search Tool) analysis.

### Re-transforming pACT2-DDX56 into the yeast cells and checking NS1 and DDX56 interaction

To eliminate false-positive interactions and check the interaction NS1 between DDX56, pACT2-DDX56 plasmid coding a fusion of GAL4-AD-DDX56 or pACT2 plasmid (as a control) were transformed into the *S. cerevisiae* strain PJ69-4A containing the NS1 bait plasmid (pGBD-NS1_W_ or pGBD-NS1_D_) or pGBD-C1 (as a control) with the LiAc/PEG. Double transformants were selected on SD agar plates (without Leu and Trp). To define the growth profiles, four colonies were cultured on SD agar plates (without Ade, His, Leu, and Trp) and tested for β-galactosidase enzyme activities.

### β-galactosidase assay

Colonies defined the growth profiles were grown in a 5 mL SD medium (without Trp/Leu or Trp) at 30 °C. The cells in 500 µL of saturated culture were recovered with centrifugation and re-suspended in 300 µL Z- buffer (0.1 M sodium phosphate pH 7.0, 10 mM KCl, 1 mM MgSO_4_, and 0.35% β-mercaptoethanol). The cells in suspensions were disintegrated in a freeze-thaw procedure repeated five times in liquid nitrogen. Then, 60 µL o-nitrophenyl-β-d-galactopyranoside (ONPG) (4 mg/mL) substrate was added and incubated for 60 minutes at 37 °C. To stop the reactions 300 µL Na_2_CO_3_ (0.5 M) was added. The supernatants were recovered by centrifugation at 15000 rpm for 5 min, and then the absorbance of samples at 420 nm (OD_420_) was measured. The β-galactosidase activities were calculated from a standard curve plotted with a recombinant β-galactosidase enzyme (Roche).

### Co-immunoprecipitation assay

Co-immunoprecipitation assays were used to confirm the interaction between NS1 and DDX56 in mammalian cells. The HEK293 cells (5x10^5^) were seeded in a 6-well plate and incubated under standard culture conditions (37 °C, 5% CO_2,_ and 90% relative humidity) for 20-24 h. The cells were co-transfected with the plasmid DNAs expressing H-DDX56 and influenza A virus NS1 protein and incubated for 48 h. Furthermore, HEK293 cells were transfected with pCHA-DDX56 plasmid and the cells were infected at 40 h post-transfection with influenza A/WSN/33/H1N1 virus at one MOI and incubated for 8 h. After the incubation periods, transfected or virus-infected cells were washed with PBS and treated with 1 mM DSP (dithiobis succinimidyl propionate) for 1 h. Cross-linking reaction was stopped by the addition of glycine at 100 mM final concentration, and then the cells were lysed in buffer A (50 mM Tris- HCl pH 8.0, 150 mM NaCl, 1 mM DTT, and 0.1% NP-40), and the lysates were clarified by centrifugation at 10000 rpm for 5 min. The cell lysates (300 µL) were incubated with 5 µL monoclonal mouse anti-HA antibody (sc-7392) at 4 °C for 2 h. Protein A Sepharose (GE Healthcare, Sweden) was added to lysates and moderately stirred overnight at 4 °C. The beads were washed three times with buffer A. The proteins bound Sepharose beads were recovered by heating the samples at 95 °C for 5 minutes in SDS-PAGE loading buffer and then analyzed by western blotting.

### Immunofluorescence assay

The localization of H-DDX56 and influenza A virus NS1 proteins in transfected HeLa cells were analyzed with an immunofluorescence assay. HeLa cells grown on coverslips were transfected with the plasmid DNAs. After 36-40 h transfection, the cells were washed three times with PBS, fixed in 3% paraformaldehyde for 20 min, and permeabilized with 0.1% NP-40. After blocking in 1% skim milk for 30 min, the cells were incubated with the primary antibodies (mouse anti-HA and/or rabbit anti-NS1) diluted in 1% skim milk for 60 min at room temperature and then washed twice with 0.1% NP-40 and once with PBS. After a second blocking with 1% skim milk for 20 min, the cells were incubated with the Alexa-488-conjugated goat anti-mouse IgG and/or Alexa-568-conjugated goat anti-rabbit IgG (at 1:300 dilutions in 1% skim milk) for 60 min. The nuclei of the cells were stained with DAPI (4’,6’-diamidino-2-phenylindole). The coverslip was washed in 0.1% NP-40 and mounted in a media (0.1% p-phenylendiamine and 80% glycerol), and the cells were analyzed using a fluorescence microscope (Olympus BH40, Japan).

### siRNA transfection and quantitative real-time PCR analysis

To understand the potential role of DDX56 on influenza A virus replication, we used RNA interference techniques. The small interfering RNA of DDX56 (siDDX56) (Cat(1299001/ HSS182589) was purchased from Life Technologies. The HeLa cells (3×10^5^) were seeded in six well-plates and incubated under standard culture conditions for 20-24 h. The cells were transfected with 30 pmol siDDX56 or nonsilencing control siRNA (Invitrogen, # 12935-200) with lipofectamine RNAiMAX (Thermo Fisher Scientific, USA) reagent according to the manufacturer’s protocol and incubated for 48 h. After the incubation, total RNA was extracted from siRNA transfected cells for quantitation of the DDX56 transcript with RT-qPCR. Remaining knock-down cells were sub-cultured for influenza A virus infection. Quantitation of the DDX56 transcript in the cells transfected with siRNA was analyzed with RT-qPCR. Total RNA and cDNAs were prepared as described above. Real-time PCR was conducted using the FastStart Universal SYBR Green Master mix (Roche, Germany). Reactions were carried out in triplicate in a total volume of 10 µL containing 2 µL of cDNA and 2.5 pM gene-speciﬁc primers. Bio-Rad, the CFX96 model real time PCR instrument, was used for quantitative analysis. The cycle conditions included an initial denaturation step at 94 °C for 5 min, followed by 45 cycles of amplification for 10 s at 94 °C, 30 s at 58 °C, and 15 s at 72 °C. Fluorescence was quantiﬁed during the 58°C annealing step, and the product formation was conﬁrmed by melting curve analysis (65 °C to 95 °C). As an internal control, the level of the housekeeping actin-beta (ACTB) gene transcript was determined. The amounts of the DDX56 were normalized by the amount of actin-beta transcripts. DDX56/831-For and DDX56/959-Rev primer pair and ACTB-For/ACTB-Rev primer pair were used for amplification of the DDX56 and ACTB cDNAs, respectively. The primer sequences used for the RT-qPCR are listed in Table 1.

### Influenza A virus growth in DDX56 knock-down cells

The quantity of M1 protein, one of the most abundant virus structural proteins in the infected cell, was analyzed by western blotting to observe influenza A virus growth in knock-down cells at early stages of replication. Forty-eight hours post-transfection with siRNAs, the cells were sub-cultured into 24-well plates (2 ×10^5^/well) and incubated for 24 h. Then the monolayer cultures were infected with human influenza A (WSN) viruses at 1MOI. After the virus adsorption at 37 °C for 30 min, the inoculum was removed, and the cells were maintained in the maintenance medium for 8 h. The cells were lysed in the SDS-PAGE sample buffer and viral structural M1 proteins in the lysates were analyzed with SDS-PAGE/Western blotting. In late stage of viral growth, the quantity of virus released into the medium in the infected culture was determined by plaque assay. For this, the knock-down cells were seeded in 12-well plates (4 ×10^5^/well), cultured for 24 h and inoculated with influenza A viruses at 0.1 MOI. The inoculum was removed, and the cultures were incubated in the maintenance medium for 24 h at 37 ºC. At 8, 12 and 24 h post infection, the medium containing released viruses was collected, and the quantity of the viruses in the medium were determined by plaque assays using MDCK cells (Turan *et al*., 1996).

### Over-expression H-DDX56 and viral infection

To understand the effect of DDX56 on influenza A virus replication, H-DDX56 protein was over-expressed by transient transfection of plasmid encoding this protein, and the effects of H-DDX56 increase on the virus replication was investigated. The HEK293 cells (2.5x10^5^) were seeded in 12-well plate and incubated under standard culture conditions for 20-24 h and the pCHA-DDX56 plasmid DNA was transfected with 2 µg. After 40 h transfection, the cells were infected with influenza A (WSN) viruses at 1MOI and incubated for 8h. Then the cells were lysed in the SDS-PAGE sample buffer. H-DDX56 and viral proteins in lysates were analyzed with SDS-PAGE/Western blotting.

### ***In silico* prediction of NS1 and DDX56 interaction**

The *in silico* method was used to predict possible interactions between influenza A virus NS1 and human DDX56 protein. The three-dimensional structure of NS1 and DDX56 was predicted by using (I-TASSER algorithm (https://zhanglab.ccmb.med.umich.edu/I-TASSER/) (Zhang, 2008). Interaction models showing the highest binding affinity between two proteins according to their free binding energies were determined by using the top 3D models of NS1 and DDX56 with pyDOCK protein-protein docking method (http://life.bsc.es/pid/pydock) (Jimenez-Garcia *et al*., 2013). Finally, the computational analysis of protein interaction domains was performed via Vega ZZ, a molecular modeling program (Pedretti *et al*., 2002).

## Results

### Cellular RNA helicase DDX56 interacts with influenza A virus NS1 protein in yeast cells

To understand the roles of NS1 in the pathogenesis of influenza A virus, we screened host proteins that interact with NS1 protein using a GAL4-based yeast two-hybrid assay. Following the two-hybrid system protocol, the pGBD-NS1_W_ plasmid or the pGBD-NS1_D_ plasmid as bait and a human cDNA library cloned into pACT2 were used. The library screening yielded approximately 150 colonies on the SD agar medium (without Ade, His, Leu, and Trp) after three days of growth at 30 °C. The number was reduced to 50 colonies by eliminating false-positive interactors after the β-galactosidase assay. Plasmids DNA carrying cDNA were isolated from the colonies with high β-galactosidase activity, and the cDNAs were sequenced. Sequencing results showed that some of the cDNA were not in the codon frame with GAL4 AD. The candidate genes within the codon frame with GAL4 AD were defined with BLAST analysis. The cDNAs were belong to genes coding DEAD (Asp-Glu-Ala-Asp) box helicase 56 (DDX56), neuroguidin, EIF4E binding protein (NGDN), proteosome subunit beta type 4 (PSMB4), ribosomal protein L29 (RPL29), and small nuclear ribonucleoprotein D1 polypeptide (SNRPD1) (Figures S1-S5). We focused on DDX56, which has been shown to be effective in intracellular replication of various viruses among these proteins (Xu *et al*., 2011; Xu and Hobman, 2012; Fu *et al*., 2019a; Fu *et al*., 2019b) by directly interacting with the viral proteins or indirectly with host proteins.

**Figure 1 -:**
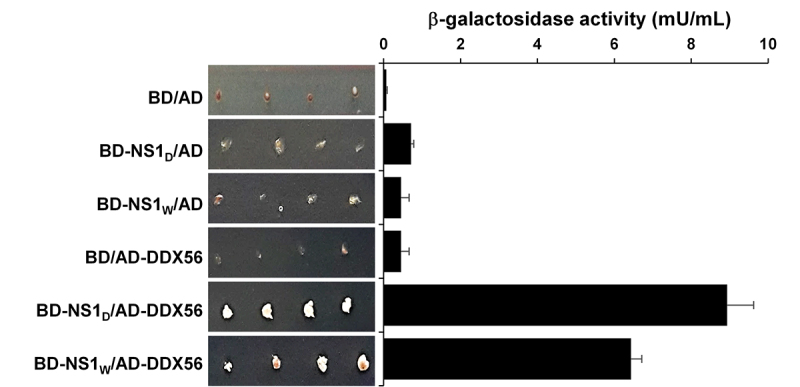
The growth profiles and β-galactosidase activity of double transformed *S. cerevisiae* strain PJ69-4A with yeast two-hybrid plasmids. The yeast cells were transformed with a plasmid coding GAL4-binding domain (BD) (pGBD-C1) or viral NS1 bait protein fused with GAL4-BD (pGBD-NS1_W_ or pGBD-NS1_D_) and then with a plasmid coding GAL4-activation domain (AD) (pACT2) or human DDX56 protein fused with GAL4-AD (pACT2-DDX56). To define the growth profiles, four colonies were cultured on SD agar plates (without Ade, His, Leu, and Trp). The β-galactosidase enzyme activities of the colonies were defined as described in Methods.

The analysis indicated that the DDX56 cDNA part of the GAL4-AD-DDX56 fusion gene consists of the sequence coding 141 amino acids residue of the carboxy-terminal part of the DDX56 protein (Figure S1). Therefore, the full size of DDX56 gene consisting of the sequence coding 548 amino acids residue was amplified with reverse transcription-PCR and cloned into the pACT2 yeast two-hybrid plasmid to construct pACT2-DDX56. To check the result of two-hybrid screening and eliminate false-positive interactions, pACT2-DDX56 plasmid encoding AD-DDX56 was re-transformed into PJ69-4A yeast strain expressing NS1_W_ or NS1_D_ bait protein, and the growth profiles of the double transformants on the SD agar medium (without Ade, His, Leu, and Trp) were determined (Figure 1). The growth of colonies in the selective SD media indicated a positive interaction between DDX56 and human or avian influenza A virus NS1 protein. As expected, the growth of colonies carrying control plasmid (pGBD-C1 or pACT2) was not observed in the selective media. In addition to growth profiles, to confirm the results, we performed β-galactosidase assay in the same colonies. The results indicated that the β-galactosidase enzyme activity significantly increased in yeast cells expressing both NS1 bait protein and DDX56 protein (Figure 1). Very low levels of β-galactosidase activities were detected in the cells transformed with plasmid expressing either NS1 bait protein or DDX56 protein used as control.

### The DDX56 interacts with influenza A virus NS1 protein in mammalian cells

**Figure 2 -:**
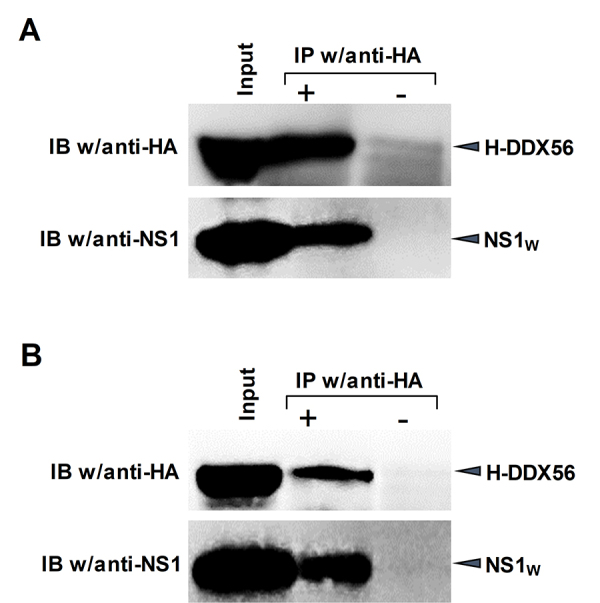
The immunoprecipitation analysis of human DDX56 and influenza A virus NS1 protein interaction in mammalian cells. The cells were co-transfected with pCHA-DDX56 plasmid and pCAGGS-NS1_w_ (**A**) or transfected with pCHA-DDX56 plasmid and infected with influenza A (WSN) viruses (**B**). The cell lysates were prepared, and H-DDX56 proteins were precipitated with mouse monoclonal anti-HA antibody as described in the methods section. For blotting rabbit polyclonal anti-NS1 and mouse monoclonal anti-HA antibody were used.

**Figure 3 -:**
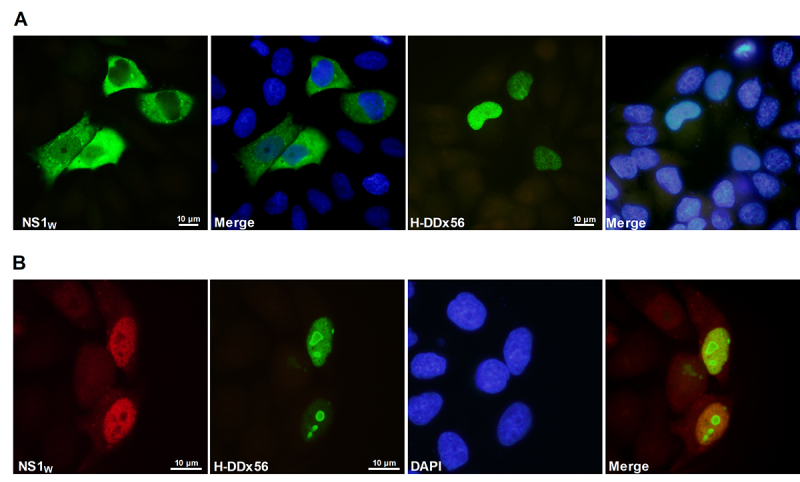
Localization patterns of influenza A virus NS1 and human DDX56 proteins in HeLa cells transfected with a single plasmid (**A**) or double plasmid DNAs (**B**). The cells were fixed with 3% paraformaldehyde and immunostained with specific antibodies after 36 hours post-transfection. As primary antibodies, mouse monoclonal anti-HA (for H-DDX56) and/or rabbit polyclonal anti-NS1 (for NS1_w_) were used; as the secondary antibodies, monoclonal anti-mouse IgG conjugated Alexa-488 (for H-DDX56) and the monoclonal anti-rabbit IgG conjugated Alexa-488 or Alexa-568 (for NS1 proteins) were used. The nuclei of the cells were stained with DAPI. The samples were examined with X100 oil immersion objective. Images were captured using a fluorescence microscope (Olympus BH40, Japan).

In order to evaluate the relationship between the influenza A virus NS1 protein and cellular RNA helicase DDX56 having interaction in yeast cells, a co-immunoprecipitation assay and an immunofluorescence assay were carried out in mammalian cells. Therefore, the HEK293 cells were co-transfected with plasmids expressing H-DDX56 protein and influenza A virus NS1 protein (NS1_W_), and the lysates of these cells were subjected to immunoprecipitation with anti-HA antibody against H-DDX56. The immunoprecipitated proteins were analyzed by SDS-PAGE /Western blotting using both mouse anti-HA and rabbit anti-NS1 antibodies. The results showed that the influenza A virus NS1 protein was co-precipitated with H-DDX56 protein. The NS1 protein was not detected in the immunoprecipitates without anti-HA antibody (Figure 2A). The co-immunoprecipitation assay was also carried out with the cells transfected with plasmid DNA coding H-DDX56 protein and then infected with influenza A (WSN). The results showed that viral NS1 protein was also co-precipitated with human DDX56 protein in virus-infected cells (Figure 2B). In order to support the relationship between viral NS1 and human DDX56 protein in the mammalian cells, the cellular localization patterns of these proteins were investigated by immunofluorescence techniques. The results showed that some human DDX56 proteins localize in the nucleus and dominantly in the nucleolus of the cells when expressed alone, while viral NS1 localizes in the cytoplasm (Figure 3A). The human DDX56 proteins localization was not changed in the cells expressing NS1_w_ proteins (Figure 3B). In contrast, viral NS1 proteins showed a tendency to co-localize with human H-DDX56 and localized predominantly in the nucleus of the cells expressing both H-DDX56 and viral NS1 proteins, which supports the IP and yeast two-hybrid assays results. 

### Effects of DDX56 on influenza virus replication

**Figure 4 -:**
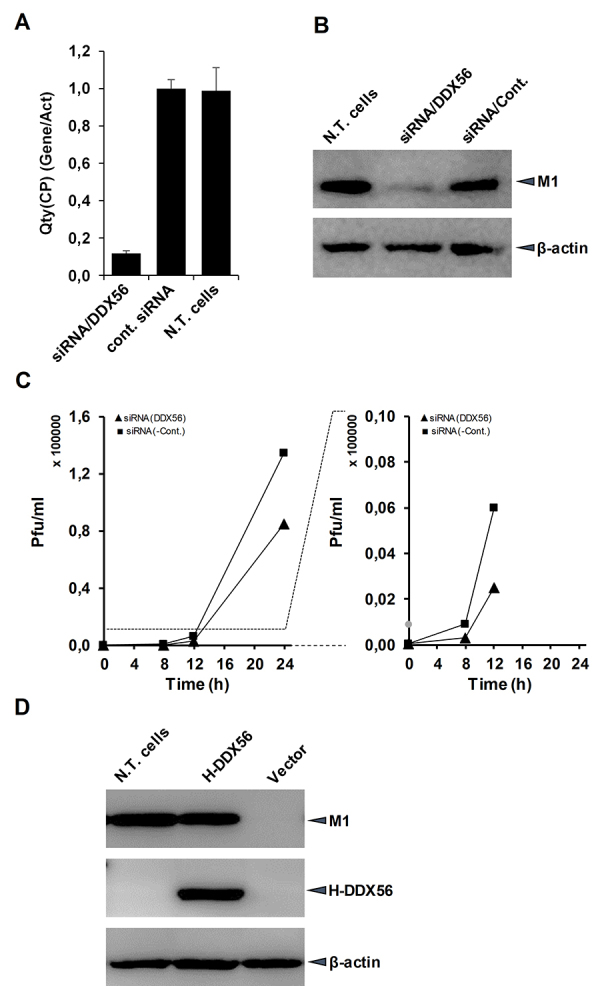
The quantitation of human DDX56 transcript and viral M1 protein in the cells transfected with siRNA or H-DDX56 encoding plasmid DNA (pCHA-DDX56) and infected with influenza A viruses. **A**. Quantity of human DDX56 transcript in the non-transfected HeLa cells (N.T. cells), the HeLa cells transfected with DDX56 specific siRNA (siRNA/DDX56), and negative control siRNA (cont.siRNA). **B**. Viral M1 protein quantity in HeLa cells transfected with siRNAs and then infected with viruses. **C.** The quantity (plaque forming unit; pfu/ml) of influenza A virus in medium of knock down/infected cell culture**. D**. The M1 protein quantity in non-transfected HEK293 cells (N.T. cells) and in HEK293 cells transfected with pCHA-DDX56 or pCHA (Vector) plasmid and then infected with human influenza A (WSN) viruses. After 8 h p.i. the cells harvested and the proteins in the cell lysates were separated on 10% PAGE. As primary antibodies, mouse monoclonal anti-HA, rabbit polyclonal anti-M1 serum, and mouse monoclonal anti-actin beta antibodies were used.

The effect of human DDX56 protein on influenza A virus replication was evaluated in knock down cells for this gene and in the cells over-expressing H-DDX56 protein with a transient transfection. The DDX56 transcript level was reduced in HeLa cells with transfection of gene-specific siRNA. The quantity of DDX56 transcript normalized with the actin beta gene was detected with RT-qPCR. The data showed that the DDX56 mRNA level was decreased by approximately 90% (Figure 4A). The knock down cells were infected with human influenza A (WSN) viruses and at an early phase of infection (8 h p.i.), and the M1 protein that is one of the most abundant viral structural proteins encoded from segment 7 were analyzed by immunoblotting. The influenza A virus M1 protein was found markedly reduced in DDX56-knockdown cells compared with that in the control cells (Figure 4B). The quantity of the viruses released in the medium in infected cultures at the later stages of infection were determined with plaque assay. At 12 and 24 h post infection, the lower titer of viruses was detected in the cells transfected with DDX56 siRNAs than that of the control cells. (Figure 4C).

In order to see the effect of DDX56 over-expression on the influenza virus replication, HEK293 cells were transfected with the plasmid encoding this protein and then infected with human influenza A (WSN) viruses. At 8 h p.i. the cells were lysed in SDS-sample buffer, and the viral M1 protein was analyzed with Western blotting. As shown in Figure 4D, the influenza A virus replication was not significantly changed in the HEK293 cells, overexpressing DDX56 compared to the control cells. The data obtained from the cells overexpressing the DDX56 and knockdown cells suggests that the DDX56 protein has a positive regulatory effect on influenza A virus replication.

### ***In silico* prediction of NS1 and DDX56 interaction**

Since the native structures are unknown, the tertiary structures of full-size human DDX56 and influenza NS1 proteins were predicted by using the I-TASSER algorithm from on line data server. The C-scores and 3D structures of the five top models are given in Table 2 and Figure S6 and S7, respectively.

**Table 2 -:** C-scores for predicted 5 top NS1 and DDX56 models.

Models	C-score	Exp.TM-Score	Exp.RMSD	No.of decoys	Cluster density
NS1-1	-0.59	0.64 ± 0.13	6.9 ± 4.1	6410	0.1134
NS1-2	-1.17			3329	0.0633
NS1-3	-1.36			2890	0.0523
NS1-4	-2.99			4465	0.0102
NS1-5	-0.80			4197	0.0918
DDX56-1	-1.05	0.58 ± 0.14	10.0 ± 4.6	1165	0.0841
DDX56-2	-1.44			741	0.0573
DDX56-3	-2.27			318	0.0249
DDX56-4	-3.22			134	0.0096
DDX56-5	-2.31			362	0.0239

**Figure 5 -:**
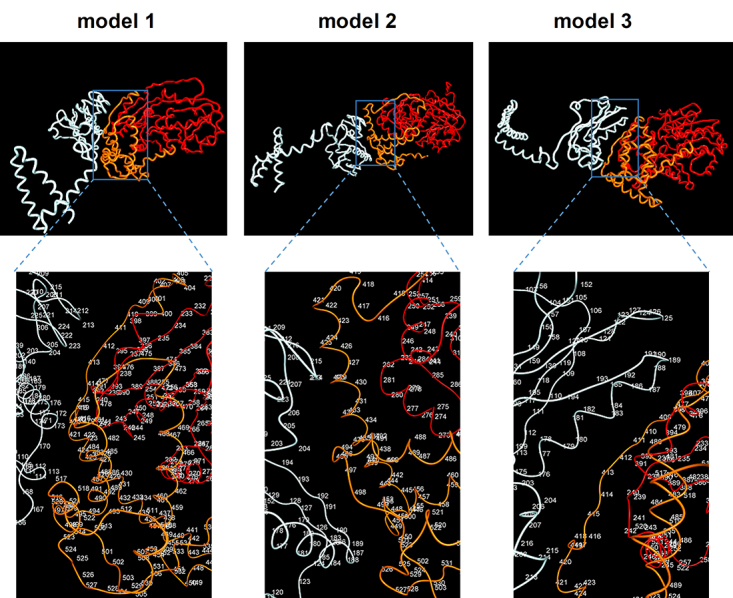
*In silico* models of the influenza A virus NS1 and human DDX56 protein interaction. The predicted ternary structures of NS1(white) and human DDX56 (red and orange) proteins are shown. In the lower panel, the predicted interface of NS1 and DDX56 proteins are given as enlarged and numbered amino acid residues. The 3D-structures and the interaction models of the proteins were obtained using I-TASSER algorithm and Pymol software, respectively. The Gibbs free energy of binding ((G) for interactions: -56.267 (model 1), -50.429 (model 2), -47.185 (model 3).

The interactions between model NS1-1 and model DDX56-1, the top models determined with the I-TASSER algorithm, were predicted with the pyDOCK protein-protein docking method (Jimenez-Garcia *et al*., 2013). Three predicted interaction models with the highest binding affinity (with lower Gibbs' free energy of binding/ΔG) were analyzed with the Vega ZZ modeling program. In these interaction models, the NS1 protein was found to bind to the carboxyl terminal region of the DDX56 protein (Figure 5). These results support the findings of the yeast two hybrid assay that showed the interaction of the carboxyl terminal domain (last 141 amino acid residues) of human DDX56 with viral NS1 protein.

## Discussion

One of the important properties of influenza A viruses that distinguishes it from the other RNA viruses is the necessity to transport viral genome components into the host cell nuclei for replication and transcription. The main reason for this is that the viral RNA polymerase enzyme is dependent on the cellular RNA polymerase II enzyme (Engelhardt *et al*., 2005). Influenza A virus RNA dependent RNA polymerase (RdRP) removes the 5′ cap structure of pre-mRNAs synthesized by RNA pol II (cap snatching activity) and uses it in the transcription of viral mRNAs (Dias *et al*., 2009). Therefore, the nucleo-cytoplasmic traffic of the influenza virus components in the host cells is much more intense compared to the other RNA viruses. Several cellular protein factors involved in influenza A virus replication and the nucleo-cytoplasmic traffic of viral components were identified (Senbas Akyazi *et al*., 2020). In order to understand the influenza A virus replication strategies, it is very important to reveal the relationship between cellular and viral factors at the molecular level. Some of the host cellular protein factors interfering with influenza A virus replication regulate the viral replication positively while others have negative effects (Turan *et al*., 2004; Gao *et al*., 2015). In this study, the new host proteins that may be related to the viral NS1 protein which functions to eliminate the host interferon response to influenza A viruses (Gao *et al*., 2012) was screened with the yeast two-hybrid method, and several candidate protein factors as NS1 interactor were determined (Figures S1-S5). Among these proteins, the relationship of human DDX56 protein (as also known NOH61) (Zirwes *et al*., 2000) with the viral NS1 protein in both yeast and mammalian cells and the effects of this protein on influenza A virus replication were investigated. The interaction between these two proteins was also evaluated with *in silico* methods. The yeast two-hybrid analysis was repeated with pACT2-DDX56 constructed by cloning the complete cDNA of DDX56. The growth profiles of double transformants were defined on the selective medium for the primary screening and then tested by the β-galactosidase assay. The results showed that both human and avian influenza A virus NS1 proteins and DDX56 protein have an interaction in yeast cells (Figure 1). 

The DDX56 protein is a member of the DEAD box protein family with ATP-dependent RNA helicase activity. All DEAD-box proteins contain a highly conserved helicase core that consists of two RecA-like domains with at least 12 conserved amino acid motifs. The helicase core has ATP binding, RNA binding, and RNA duplex unwinding activities (Linder *et al*., 1989; Cordin *et al*., 2006). The DEAD-box helicases are identified as 37 members in human and 26 in *S. cerevisiae*. One of them, the human DDX56 (yeast homologue Dbp9), mainly functions during ribosome biogenesis and the assembly of 60S ribosomal subunits in the nucleolus as well as in all biological processes where RNA plays a central role such as transcription, mRNA splicing, mRNA transport, translation, and mitochondrial gene expression. Recent studies described how RNA helicases participate in viral infections. Some RNA viruses carry their own helicases and use them for a replication process such as HCV NS3. Modulating the nuclear-cytoplasmic transport is essential for HCV replication (Gu and Rice, 2010). On the other hand, most RNA viruses do not encode their own helicase and use the cellular RNA helicases to support their life cycle, such as HIV-I, WNV, and IAV. Some cellular helicases enhance viral replication like a co-factor. During HIV-I infection, DDX1 and DDX5/p68 enhance the Rev-mediated nuclear export of HIV-1 transcripts (Yasuda-Inoue *et al*., 2013). In contrast, some cellular helicases play central roles in the antiviral defense mechanism. During HBV infection, DDX3 restricts viral replication by binding to HBV polymerase and inhibiting reverse transcription (Wang H *et al*., 2009). Therefore, we speculated that the cellular RNA helicase DDX56 could affect influenza A virus replication by interacting with viral NS1 protein in infection.

The yeast two-hybrid method is an effective technique in determining the relationship between proteins and protein functions, but it is an artificial system and may not fully reflect the native conditions. Therefore, the relationship between influenza A virus NS1 and human DDX56 proteins and the effects of DDX56 protein on influenza virus replication were evaluated from mammary cells. The open reading frames of human DDX56 and influenza A virus NS1 genes were cloned in the mammalian expression plasmids (pCAGGS or pCHA), and the plasmid vectors were used for transient transfection of HEK293 and HeLa cells. The immunoprecipitation and immunofluorescence techniques were applied to reveal the relationship between human DDX56 and influenza A virus NS1 protein synthesized in virus infected and/or transiently transfected cells. For the co-immunoprecipitation assays, HEK293 cells were co-transfected with plasmids expressing H-DDX56 protein and influenza A virus NS1 protein. We also tested whether viral NS1 protein could interact with DDX56 protein in virus infected cells. For this aim, HEK293 cells were transfected with pCHA-DDX56 plasmid vector and then infected with influenza A (WSN) viruses. The cell lysates were subjected to co-IP with anti-HA antibody. The results showed that the viral NS1 protein co-precipitated with H-DDX56, suggesting that human DDX56 and influenza A virus NS1 protein interact in mammalian cells (Figure 2). The intracellular localization patterns of the proteins also give important clues about the interaction between proteins. The co-localization tendency of viral NS1 proteins with H-DDX56 protein in the nuclei of transiently transfected HeLa cells supports the IP results (Figure 3).

In several reports, although not related with the influenza A viruses, it was shown that RNA helicase DDX56 interferes with some virus replication. For instance, DDX56 has an up-regulator effect on FMDV replication and co-expression DDX56, and FMDV 3A protein inhibits the type I interferon by reducing the phosphorylation of IRF3 (Fu *et al*., 2019a). On the other hand, DDX56 is not important for the replication of WNV. Instead, DDX56 interacts with WNV capsid protein and plays an essential role in the assembly of infectious WNV virions (Xu *et al*., 2011; Xu and Hobman, 2012). Furthermore, during WNV infection, the nucleolar helicase DDX56 changes localization to virus assembly sites on the endoplasmic reticulum (Reid and Hobman, 2017). In addition to DDX3 and DDX5, DDX56 enhances Rev-dependent RNA export function of the unspliced/partially spliced HIV-1 mRNAs during HIV-I infection (Yasuda-Inoue *et al*., 2013).

In this study, the interaction of influenza A virus NS1 and human DDX56 proteins, both in yeast and mammalian cells, was shown. In order to reveal the effects of DDX56 protein on influenza A viruses, the viral replication in the cells knocked down with specific siRNA for DDX56 transcript or over-expressed DDX56 protein was monitored with immunoblotting. The influenza A virus replication was downregulated in the cells transfected with DDX56 specific siRNA (Figure 4B and 4C), showing that the DDX56 proteins have stimulatory effects on influenza A virus replication. In contrast, no significant changes were observed in the quantity of viral M1 proteins in the virus infected cells over-expressing DDX56 (Figure 4D). From this result, it was concluded that the endogenous DDX56 protein are sufficient for viral replication. The significant decrease of viral M1 protein in virus infected knock down cells indicates that DDX56 protein has a positive regulatory effect on influenza A virus replication. However, the action mechanism of DDX56 on virus replication and the role of NS1 in this event are not clearly understood. It has been reported that DDX56 has a negative regulatory effect on virus-triggered type I IFN induction by disrupting the interaction between IRF3 and IPO5, which inhibits the nuclear translocation of IRF3 (Li *et al*., 2020). The positive regulator effect of DDX56 on influenza A virus replication may be due to the disruption of IFN induction. Another speculation to explain the effect of DDX56 on viral replication is that DDX56 and NS1 may cooperate for remodeling of the secondary structure of viral RNAs and enhance the viral RNA polymerase activity.

In the yeast cells, the carboxyl terminal region consisting of 141 amino acid residues of DDX56 protein fused with GAL4 AD interacts with viral NS1 protein (Figure S1). *In silico* predicted protein interface models also support the binding of DDX56 protein to NS1 protein via carboxyl terminal region (Figure 5). From these results, it was concluded that the carboxyl terminal region of the DDX56 is important for interaction with NS1 protein. However, the action mechanism of human DDX56 on influenza A virus replication requires further elucidation.

In conclusion, it has been shown that the human DDX56 protein, ATP-dependent RNA helicase, interacts with influenza A virus NS1 protein in both yeast and mammalian cells and has a positive regulatory effect on viral replication. 

## Supplementary material

The following online material is available for this article:

Figure S1 - The sequencing chromatogram (A) and BLAST analysis (B) of DEAD (Asp-Glu-Ala-Asp) box helicase 56 (DDX56).

Figure S2 - The sequencing chromatogram (A) and BLAST analysis (B) of neuroguidin, EIF4E binding protein (NGDN).

Figure S3 - The sequencing chromatogram (A) and BLAST analysis (B) of proteasome subunit beta type 4 (PSMB4).

Figure S4 - The sequencing chromatogram (A) and BLAST analysis (B) of ribosomal protein L29 (RPL29).

Figure S5 - The sequencing chromatogram (A) and BLAST analysis (B) of small nuclear ribonucleoprotein D1 polypeptide (SNRPD1).

Figure S6 - The top five I-TASSER 3D models of human DDX56 protein.

Figure S7 - The top five I-TASSER 3D models of influenza A virus NS1 protein.

## References

[B1] Basu D, Walkiewicz MP, Frieman M, Baric RS, Auble DT, Engel DA (2009). Novel influenza virus NS1 antagonists block replication and
restore innate immune function. J Virol.

[B2] Burgui I, Aragon T, Ortin J, Nieto A (2003). PABP1 and eIF4GI associate with influenza virus NS1 protein in
viral mRNA translation initiation complexes. J Gen Virol.

[B3] Cordin O, Banroques J, Tanner NK, Linder P (2006). The DEAD-box protein family of RNA helicases. Gene.

[B4] de la Luna S, Fortes P, Beloso A, Ortin J (1995). Influenza virus NS1 protein enhances the rate of translation
initiation of viral mRNAs. J Virol.

[B5] Dias A, Bouvier D, Crepin T, McCarthy AA, Hart DJ, Baudin F, Cusack S, Ruigrok RW (2009). The cap-snatching endonuclease of influenza virus polymerase
resides in the PA subunit. Nature.

[B6] Engelhardt OG, Smith M, Fodor E (2005). Association of the influenza A virus RNA-dependent RNA polymerase
with cellular RNA polymerase II. J Virol.

[B7] Fu SZ, Yang WP, Ru Y, Zhang KS, Wang Y, Liu XT, Li D, Zheng HX (2019). DDX56 cooperates with FMDV 3A to enhance FMDV replication by
inhibiting the phosphorylation of IRF3. Cell Signal.

[B8] Fu SZ, Li L, Zhang J, Li D, Zheng HX (2019). Porcine DDX56 regulates the Foot and Mouth disease virus
replication and the virus-triggered RLR pathway. Acta Vet Zoot Sinica.

[B9] Gao S, Song L, Li J, Zhang Z, Peng H, Jiang W, Wang Q, Kang T, Chen S, Huang W (2012). Influenza A virus-encoded NS1 virulence factor protein inhibits
innate immune response by targeting IKK. Cell Microbiol.

[B10] Gao S, Wu J, Liu RY, Li J, Song L, Teng Y, Sheng C, Liu D, Yao C, Chen H (2015). Interaction of NS2 with AIMP2 facilitates the switch from
ubiquitination to SUMOylation of M1 in influenza A virus-infected
cells. J Virol.

[B11] Gu M, Rice CM (2010). Three conformational snapshots of the hepatitis C virus NS3
helicase reveal a ratchet translocation mechanism. Proc Natl Acad Sci U S A.

[B12] Hale BG, Randall RE, Ortin J, Jackson D (2008). The multifunctional NS1 protein of influenza A
viruses. J Gen Virol.

[B13] Hatada E, Fukuda R (1992). Binding of influenza A virus NS1 protein to dsRNA in
vitro. J Gen Virol.

[B14] James P, Halladay J, Craig EA (1996). Genomic libraries and a host strain designed for highly efficient
two-hybrid selection in yeast. Genetics.

[B15] Jimenez-Garcia B, Pons C, Fernandez-Recio J (2013). pyDockWEB: a web server for rigid-body protein-protein docking
using electrostatics and desolvation scoring. Bioinformatics.

[B16] Kawaoka Y, Neumann G (2012). Influenza viruses: an introduction. Methods Mol Biol.

[B17] Kochs G, Garcia-Sastre A, Martinez-Sobrido L (2007). Multiple anti-interferon actions of the influenza A virus NS1
protein. J Virol.

[B18] Lam WY, Yeung AC, Chan PK (2011). Apoptosis, cytokine and chemokine induction by non-structural 1
(NS1) proteins encoded by different influenza subtypes. Virol J.

[B19] Lamb RA, Choppin PW (1979). Segment 8 of the influenza virus genome is unique in coding for
two polypeptides. Proc Natl Acad Sci U S A.

[B20] Li D, Fu S, Wu Z, Yang W, Ru Y, Shu H, Liu X, Zheng H (2020). Correction: DDX56 inhibits type I interferon by disrupting
assembly of IRF3-IPO5 to inhibit IRF3 nucleus import. J Cell Sci.

[B21] Lin D, Lan J, Zhang Z (2007). Structure and function of the NS1 protein of influenza A
virus. Acta Biochim Biophys Sin (Shanghai).

[B22] Linder P, Lasko PF, Ashburner M, Leroy P, Nielsen PJ, Nishi K, Schnier J, Slonimski PP (1989). Birth of the D-E-A-D box. Nature.

[B23] Nagata K, Saito S, Okuwaki M, Kawase H, Furuya A, Kusano A, Hanai N, Okuda A, Kikuchi A (1998). Cellular localization and expression of template-activating
factor I in different cell types. Exp Cell Res.

[B24] Nakajima K (1997). Influenza virus genome structure and encoded
proteins. Nihon Rinsho.

[B25] Nemeroff ME, Barabino SM, Li Y, Keller W, Krug RM (1998). Influenza virus NS1 protein interacts with the cellular 30 kDa
subunit of CPSF and inhibits 3'end formation of cellular
pre-mRNAs. Mol Cell.

[B26] Neumann G, Hughes MT, Kawaoka Y (2000). Influenza A virus NS2 protein mediates vRNP nuclear export
through NES-independent interaction with hCRM1. EMBO J.

[B27] Newby CM, Sabin L, Pekosz A (2007). The RNA binding domain of influenza A virus NS1 protein affects
secretion of tumor necrosis factor alpha, interleukin-6, and interferon in
primary murine tracheal epithelial cells. J Virol.

[B28] Niwa H, Yamamura K, Miyazaki J (1991). Efficient selection for high-expression transfectants with a
novel eukaryotic vector. Gene.

[B29] Qiu Y, Nemeroff M, Krug RM (1995). The influenza virus NS1 protein binds to a specific region in
human U6 snRNA and inhibits U6-U2 and U6-U4 snRNA interactions during
splicing. RNA.

[B30] Pedretti A, Villa L, Vistoli G (2002). VEGA: a versatile program to convert, handle and visualize
molecular structure on Windows-based PCs. J Mol Graph Model.

[B31] Reid CR, Hobman TC (2017). The nucleolar helicase DDX56 redistributes to West Nile virus
assembly sites. Virology.

[B32] Senbas Akyazi B, Pirincal A, Kawaguchi A, Nagata K, Turan K (2020). Interaction of influenza A virus NS2/NEP protein with the
amino-terminal part of Nup214. Turk J Biol.

[B33] Stasakova J, Ferko B, Kittel C, Sereinig S, Romanova J, Katinger H, Egorov A (2005). Influenza A mutant viruses with altered NS1 protein function
provoke caspase-1 activation in primary human macrophages, resulting in fast
apoptosis and release of high levels of interleukins 1beta and
18. J Gen Virol.

[B34] Turan K, Nagata K, Kuru A (1996). Antiviral effect of Sanicula europaea L. leaves extract on
influenza virus-infected cells. Biochem Biophys Res Commun.

[B35] Turan K, Mibayashi M, Sugiyama K, Saito S, Numajiri A, Nagata K (2004). Nuclear MxA proteins form a complex with influenza virus NP and
inhibit the transcription of the engineered influenza virus
genome. Nucleic Acids Res.

[B36] Wang H, Kim S, Ryu WS (2009). DDX3 DEAD-Box RNA helicase inhibits hepatitis B virus reverse
transcription by incorporation into nucleocapsids. J Virol.

[B37] Wang W, Riedel K, Lynch P, Chien CY, Montelione GT, Krug RM (1999). RNA binding by the novel helical domain of the influenza virus
NS1 protein requires its dimer structure and a small number of specific
basic amino acids. RNA.

[B38] Xia S, Monzingo AF, Robertus JD (2009). Structure of NS1A effector domain from the influenza A/Udorn/72
virus. Acta Crystallogr D Biol Crystallogr.

[B39] Xu Z, Hobman TC (2012). The helicase activity of DDX56 is required for its role in
assembly of infectious West Nile virus particles. Virology.

[B40] Xu Z, Anderson R, Hobman TC (2011). The capsid-binding nucleolar helicase DDX56 is important for
infectivity of West Nile virus. J Virol.

[B41] Yasuda-Inoue M, Kuroki M, Ariumi Y (2013). Distinct DDX DEAD-box RNA helicases cooperate to modulate the
HIV-1 Rev function. Biochem Biophys Res Commun.

[B42] Zhang Y (2008). I-TASSER server for protein 3D structure
prediction. BMC Bioinformatics.

[B43] Zirwes RF, Eilbracht J, Kneissel S, Schmidt-Zachmann MS (2000). A novel helicase-type protein in the nucleolus: protein
NOH61. Mol Biol Cell.

