# Supplementary material for: Human DDX56 protein interacts with influenza A virus NS1 protein and stimulates the virus replication
Source: Genet Mol Biol. 2021 Mar 22;44(1):e20200158. doi: 10.1590/1678-4685-GMB-2020-0158 (PMC7983190; doi:10.1590/1678-4685-GMB-2020-0158)
Supplement: Figure S1 - [file 1415-4757-GMB-44-1-e20200158-s1.pdf]

Genetics and Molecular Biology

https://doi.org/10.1590/1678-4685-GMB-2020-0158

"Supplementary Material to "Human DDX56 Protein Interacts with Influenza A Virus NS1 Protein and Stimulates the Virus Replication"

Figure S1 - The sequencing chromatogram (A) and BLAST analysis (B) of DEAD (Asp-Glu-Ala-Asp) box helicase 56 (DDX56).

A

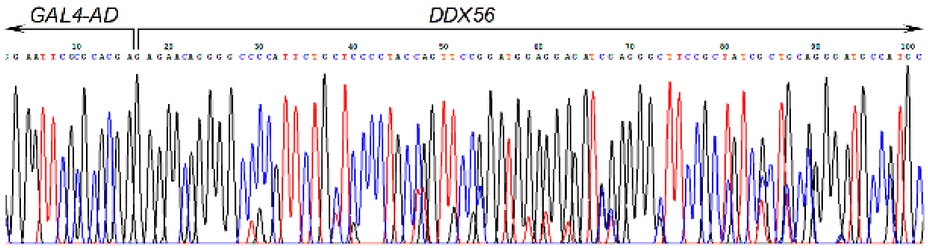

B

31.10.2020 NCBI Blast:Nucleotide Sequence

Homo sapiens DEAD-box helicase 56 (DDX56), transcript variant 1, mRNA
Sequence ID: **NM_019082.4** Length: 1855 Number of Matches: 1
Range 1: 1230 to 1854

Score	Expect	Identities	Gaps	Strand	Frame
1138 bits(616)	0.0()	622/625(99%)	0/625(0%)	Plus/Plus	

```
Query  15    GAGAGAACAGGGGCCCCATTCTGCTCCCCTACCAGTTCCGGATGGAGGAGATCGAGGGCT  74
             ||||||||||||||||||||||||||||||||||||||||||||||||||||||||||||
Sbjct  1230  GAGAGAACAGGGGCCCCATTCTGCTCCCCTACCAGTTCCGGATGGAGGAGATCGAGGGCT  1289

Query  75    TCCGCTATCGCTGCAGGGATGCCATGCGCTCAGTGACTAAGCAGGCCATTCGGGAGGCAA  134
             ||||||||||||||||||||||||||||||||||||||||||||||||||||||||||||
Sbjct  1290  TCCGCTATCGCTGCAGGGATGCCATGCGCTCAGTGACTAAGCAGGCCATTCGGGAGGCAA  1349

Query  135   GATTGAAGGAGATCAAGGAAGAGCTTCTGCATTCTGAGAAGCTTAAGACATACTTTGAAG  194
             ||||||||||||||||||||||||||||||||||||||||||||||||||||||||||||
Sbjct  1350  GATTGAAGGAGATCAAGGAAGAGCTTCTGCATTCTGAGAAGCTTAAGACATACTTTGAAG  1409

Query  195   ACAACCCTAGGGACCTCCAGCTGCTGCGGCATGACCTACCTTTGCACCCCGCAGTGGTGA  254
             ||||||||||||||||||||||||||||||||||||||||||||||||||||||||||||
Sbjct  1410  ACAACCCTAGGGACCTCCAGCTGCTGCGGCATGACCTACCTTTGCACCCCGCAGTGGTGA  1469

Query  255   AGCCCCACCTGGGCCATGTTCCTGACTACCTGGTTCCTCCTGCTCTCCGTGGCCTGGTAC  314
             |||||||||||||||||||||||||||||||||||||||||||||||||||||||||| |
Sbjct  1470  AGCCCCACCTGGGCCATGTTCCTGACTACCTGGTTCCTCCTGCTCTCCGTGGCCTGGTGC  1529

Query  315   GCCCTCCCAAGAAGCGGAAGAAGCTGTCTTCCTCTTGTAGGAAGGCCAAGAGAGCAAAGT  374
             |||||| |||||||||||||||||||||||||||||||||||||||||||||||||||||
Sbjct  1530  GCCCTCACAAGAAGCGGAAGAAGCTGTCTTCCTCTTGTAGGAAGGCCAAGAGAGCAAAGT  1589

Query  375   CCCAGAACCCACTGCGCAGCTTCAAGCACAAAGGAAAGAAATTCAGACCCACAGCCAAGC  434
             ||||||||||||||||||||||||||||||||||||||||||||||||||||||||||||
Sbjct  1590  CCCAGAACCCACTGCGCAGCTTCAAGCACAAAGGAAAGAAATTCAGACCCACAGCCAAGC  1649

Query  435   CCTCCTGAGGTTGTTGGGCCTCTCTGGAGCTGAGCACATTGTGGAGCACAGGCTTACACC  494
             ||||||||||||||||||||||||||||||||||||||||||||||||||||||||||||
Sbjct  1650  CCTCCTGAGGTTGTTGGGCCTCTCTGGAGCTGAGCACATTGTGGAGCACAGGCTTACACC  1709
```

The sequencing chromatogram (A) and BLAST analysis (B) of DEAD (Asp-Glu-Ala-Asp) box helicase 56 (DDX56). The cDNA sequence of plasmid DNA isolated from yeast cells selected with two-hybrid assay was applied to BLAST analysis provided by the NCBI.
