# Supplementary material for: Human DDX56 protein interacts with influenza A virus NS1 protein and stimulates the virus replication
Source: Genet Mol Biol. 2021 Mar 22;44(1):e20200158. doi: 10.1590/1678-4685-GMB-2020-0158 (PMC7983190; doi:10.1590/1678-4685-GMB-2020-0158)
Supplement: Figure S2 - [file 1415-4757-GMB-44-1-e20200158-s2.pdf]

Genetics and Molecular Biology

https://doi.org/10.1590/1678-4685-GMB-2020-0158

“Supplementary Material to “Human DDX56 Protein Interacts with Influenza A Virus NS1 Protein and Stimulates the Virus Replication”

Figure S2 - The sequencing chromatogram (A) and BLAST analysis (B) of neuroguidin, EIF4E binding protein (NGDN).

A

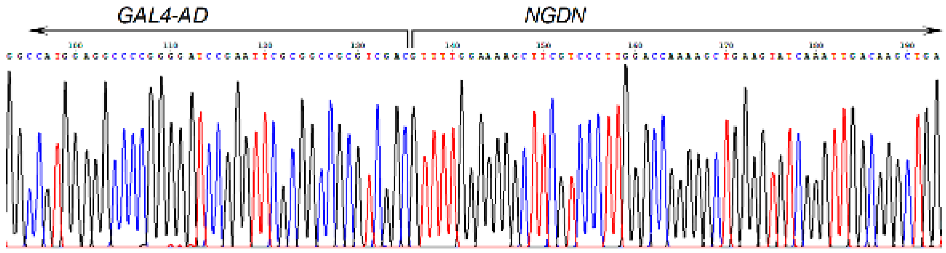

B

31.10.2020 NCBI Blast:Nucleotide Sequence

Homo sapiens neuroguidin (NGDN), transcript variant 1, mRNA
Sequence ID: **NM_001042635.2** Length: 1108 Number of Matches: 1
Range 1: 289 to 1108

Score	Expect	Identities	Gaps	Strand	Frame
1496 bits(810)	0.0()	817/820(99%)	2/820(0%)	Plus/Plus	

```
Query  131   TCG-AC-GTTTTGGAAAAGCTTCGTCCCTTGGACCAAAAGCTGAAGTATCAAATTGACAA  188
             ||| || |||||||||||||||||||||||||||||||||||||||||||||||||||||
Sbjct  289   TCGCACGGTTTTGGAAAAGCTTCGTCCCTTGGACCAAAAGCTGAAGTATCAAATTGACAA  348

Query  189   GCTGATCAAGACTGCAGTGACAGGCAGCCTTAGTGAGAATGACCCACTTCGTTTTAAGCC  248
             ||||||||||||||||||||||||||||||||||||||||||||||||||||||||||||
Sbjct  349   GCTGATCAAGACTGCAGTGACAGGCAGCCTTAGTGAGAATGACCCACTTCGTTTTAAGCC  408

Query  249   TCATCCCAGCAATATGATGAGCAAGTTGAGCTCTGAGGATGAGGAGGAAGATGAAGCAGA  308
             ||||||||||||||||||||||||||||||||||||||||||||||||||||||||||||
Sbjct  409   TCATCCCAGCAATATGATGAGCAAGTTGAGCTCTGAGGATGAGGAGGAAGATGAAGCAGA  468

Query  309   AGATGACCAGTCTGAGGCTTCAGGGAAGAAATCTGTGAAGGGAGTGTCTAAGAAATATGT  368
             ||||||||||||||||||||||||||||||||||||||||||||||||||||||||||||
Sbjct  469   AGATGACCAGTCTGAGGCTTCAGGGAAGAAATCTGTGAAGGGAGTGTCTAAGAAATATGT  528

Query  369   TCCTCCACGCTTGGTTCCAGTACATTATGATGAAACAGAAGCTGAGCGGGAGAAGAAGCG  428
             ||||||||||||||||||||||||||||||||||||||||||||||||||||||||||||
Sbjct  529   TCCTCCACGCTTGGTTCCAGTACATTATGATGAAACAGAAGCTGAGCGGGAGAAGAAGCG  588

Query  429   TCTAGAACGAGCCAAGAGACGGGCATTGAGCAGCTCTGTCATTCGTGAACTTAAGGAGCA  488
             ||||||||||||||||||||||||||||||||||||||||||||||||||||||||||||
Sbjct  589   TCTAGAACGAGCCAAGAGACGGGCATTGAGCAGCTCTGTCATTCGTGAACTTAAGGAGCA  648

Query  489   GTACTCAGATGCTCCAGAGGAAATCCGTGATGCTCGGCATCCCCATGTTACCCGCCAGAG  548
             ||||||||||||||||||||||||||||||||||||||||||||||||||||||||||||
Sbjct  649   GTACTCAGATGCTCCAGAGGAAATCCGTGATGCTCGGCATCCCCATGTTACCCGCCAGAG  708

Query  549   TCAGGAGGACCAACACAGGATTAACTATGAGGAGAGCATGATGGTGCGTTTGAGCGTCAG  608
             ||||||||||||||||||||||||||||||||||||||||||||||||||||||||||||
Sbjct  709   TCAGGAGGACCAACACAGGATTAACTATGAGGAGAGCATGATGGTGCGTTTGAGCGTCAG  768
```

The sequencing chromatogram (A) and BLAST analysis (B) of neuroguidin, EIF4E binding protein (NGDN). The cDNA sequence of plasmid DNA isolated from yeast cells selected with two-hybrid assay was applied to BLAST analysis provided by the NCBI.
