# Supplementary material for: Human DDX56 protein interacts with influenza A virus NS1 protein and stimulates the virus replication
Source: Genet Mol Biol. 2021 Mar 22;44(1):e20200158. doi: 10.1590/1678-4685-GMB-2020-0158 (PMC7983190; doi:10.1590/1678-4685-GMB-2020-0158)
Supplement: Figure S4 - [file 1415-4757-GMB-44-1-e20200158-s4.pdf]

Genetics and Molecular Biology

https://doi.org/10.1590/1678-4685-GMB-2020-0158

“Supplementary Material to “Human DDX56 Protein Interacts with Influenza A Virus NS1 Protein and Stimulates the Virus Replication”

Figure S4 - The sequencing chromatogram (A) and BLAST analysis (B) of ribosomal protein L29 (RPL29).

A

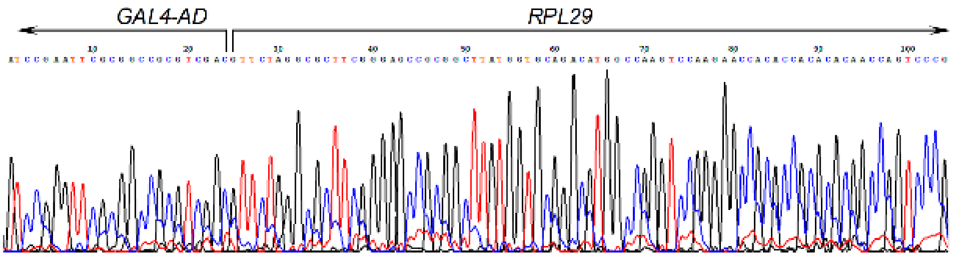

B

31.10.2020 NCBI Blast:Nucleotide Sequence

Homo sapiens ribosomal protein L29 (RPL29), mRNA

Sequence ID: **NM_000992.3** Length: 754 Number of Matches: 1

Range 1: 9 to 648

Score	Expect	Identities	Gaps	Strand	Frame
1138 bits(616)	0.0()	638/647(99%)	8/647(1%)	Plus/Plus	

```
Query  25   GTTCTAGGCGCTTCGGGAGCCGCGGCTTATGGTGCAGACATGGCCAAGTCCAAGAACCAC  84
            ||||||||||||||||||||||||||||||||||||||||||||||||||||||||||||
Sbjct  9    GTTCTAGGCGCTTCGGGAGCCGCGGCTTATGGTGCAGACATGGCCAAGTCCAAGAACCAC  68

Query  85   ACCACACACAACCAGTCCCGAAAATGGCACAGAAATGGTATCAAGAAACCCCGATCACAA  144
            ||||||||||||||||||||||||||||||||||||||||||||||||||||||||||||
Sbjct  69   ACCACACACAACCAGTCCCGAAAATGGCACAGAAATGGTATCAAGAAACCCCGATCACAA  128

Query  145  AGATACGAATCTCTTAAGGGGGTGGACCCCAAGTTCCTGAGGAACATGCGCTTTGCCAAG  204
            ||||||||||||||||||||||||||||||||||||||||||||||||||||||||||||
Sbjct  129  AGATACGAATCTCTTAAGGGGGTGGACCCCAAGTTCCTGAGGAACATGCGCTTTGCCAAG  188

Query  205  AAGCACAACAAAAAGGGCCTAAAGAAGATGCAGGCCAACAATGCCAAGGCCATGAGTGCA  264
            ||||||||||||||||||||||||||||||||||||||||||||||||||||||||||||
Sbjct  189  AAGCACAACAAAAAGGGCCTAAAGAAGATGCAGGCCAACAATGCCAAGGCCATGAGTGCA  248

Query  265  CGTGCCGAGGCTATCAAGGCCCTCGTAAAGCCCAAGGAGGTTAAGCCCAAGATCCCAAAG  324
            ||||||||||||||||||||||||||||||||||||||||||||||||||||||||||||
Sbjct  249  CGTGCCGAGGCTATCAAGGCCCTCGTAAAGCCCAAGGAGGTTAAGCCCAAGATCCCAAAG  308

Query  325  GGTGTCAGCCGCAAGCTCGATCGACTTGCCTACATTGCCCACCCCAAGCTTGGGAAGCGT  384
            ||||||||||||||||||||||||||||||||||||||||||||||||||||||||||||
Sbjct  309  GGTGTCAGCCGCAAGCTCGATCGACTTGCCTACATTGCCCACCCCAAGCTTGGGAAGCGT  368

Query  385  GCTCGTGCCCGTATTgccaaggggctcaggctgtgccggccaaaggccaaggccaaggcc  444
            ||||||||||||||||||||||||||||||||||||||||||      ||||||||||||
Sbjct  369  GCTCGTGCCCGTATTGCCAAGGGGCTCAGGCTGTGCCGGCCA------AAGGCCAAGGCC  422

Query  445  aaggccaaggccaaggatcaaaccaaggcccaggcTGCAGCCCCAGCTTCAGTTCCAGCT  504
            ||||||||||||||||||||||||||||||||||||||||||||||||||||||||||||
Sbjct  423  AAGGCCAAGGCCAAGGATCAAACCAAGGCCCAGGCTGCAGCCCCAGCTTCAGTTCCAGCT  482
```

The sequencing chromatogram (A) and BLAST analysis (B) of ribosomal protein L29 (RPL29). The cDNA sequence of plasmid DNA isolated from yeast cells selected with a two-hybrid assay was applied to BLAST analysis provided by the NCBI.
