# Supplementary material for: Human DDX56 protein interacts with influenza A virus NS1 protein and stimulates the virus replication
Source: Genet Mol Biol. 2021 Mar 22;44(1):e20200158. doi: 10.1590/1678-4685-GMB-2020-0158 (PMC7983190; doi:10.1590/1678-4685-GMB-2020-0158)
Supplement: Figure S5 - [file 1415-4757-GMB-44-1-e20200158-s5.pdf]

Genetics and Molecular Biology

https://doi.org/10.1590/1678-4685-GMB-2020-0158

"Supplementary Material to "Human DDX56 Protein Interacts with Influenza A Virus NS1 Protein and Stimulates the Virus Replication"

Figure S5 - The sequencing chromatogram (A) and BLAST analysis (B) of small nuclear ribonucleoprotein D1 polypeptide (SNRPD1).

A

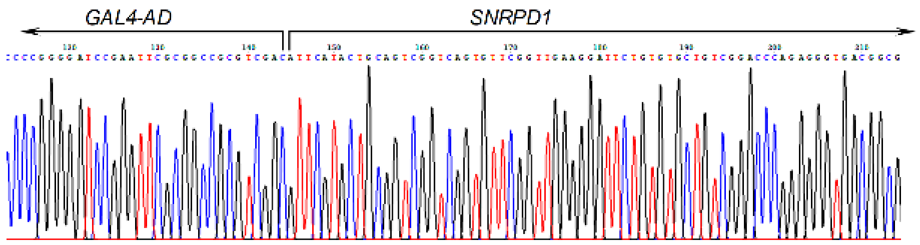

B

31.10.2020 NCBI Blast:Nucleotide Sequence

Homo sapiens small nuclear ribonucleoprotein D1 polypeptide (SNRPD1), transcript variant 1, mRN
Sequence ID: **NM_006938.4** Length: 4858 Number of Matches: 1
Range 1: 38 to 1235

Score	Expect	Identities	Gaps	Strand	Frame
1964 bits(1063)	0.0()	1156/1200(96%)	12/1200(1%)	Plus/Plus	

```
Query  144   CATTCATACTGCAGTCGGTCAGTGTTCGGTTGAAGGATTCTGTGTGCTGTCGGACCCAGA  203
             ||||||||||||||||||||||||||||||||||||||||||||||||||||||||||||
Sbjct  38    CATTCATACTGCAGTCGGTCAGTGTTCGGTTGAAGGATTCTGTGTGCTGTCGGACCCAGA  97

Query  204   GGGTGACGGCGCCGCTAGGATGAAGCTCGTGAGATTTTTGATGAAATTGAGTCATGAAAC  263
             ||||||||||||||||||||||||||||||||||||||||||||||||||||||||||||
Sbjct  98    GGGTGACGGCGCCGCTAGGATGAAGCTCGTGAGATTTTTGATGAAATTGAGTCATGAAAC  157

Query  264   TGTAACCATTGAATTGAAGAACGGAACACAGGTCCATGGAACAATCACAGGTGTGGATGT  323
             ||||||||||||||||||||||||||||||||||||||||||||||||||||||||||||
Sbjct  158   TGTAACCATTGAATTGAAGAACGGAACACAGGTCCATGGAACAATCACAGGTGTGGATGT  217

Query  324   CAGCATGAATACACATCTTAAAGCTGTGAAAATGACCCTGAAGAACAGAGAACCTGTACA  383
             ||||||||||||||||||||||||||||||||||||||||||||||||||||||||||||
Sbjct  218   CAGCATGAATACACATCTTAAAGCTGTGAAAATGACCCTGAAGAACAGAGAACCTGTACA  277

Query  384   GCTGGAAACGCTGAGTATTCGAGGAAATAACATTCGGTATTTTATTCTACCAGACAGTTT  443
             ||||||||||||||||||||||||||||||||||||||||||||||||||||||||||||
Sbjct  278   GCTGGAAACGCTGAGTATTCGAGGAAATAACATTCGGTATTTTATTCTACCAGACAGTTT  337

Query  444   ACCTCTGGATACACTACTTGTGGATGTTGAACCTAAGGTGAAATCTAAGAAAAGGGAAGC  503
             ||||||||||||||||||||||||||||||||||||||||||||||||||||||||||||
Sbjct  338   ACCTCTGGATACACTACTTGTGGATGTTGAACCTAAGGTGAAATCTAAGAAAAGGGAAGC  397

Query  504   TGTTGCaggaagaggcagaggaagaggaagaggaagaggacgtggccgtggcagaggaag  563
             ||||||||||||||||||||||||||||||||||||||||||||||||||||||||||||
Sbjct  398   TGTTGCAGGAAGAGGCAGAGGAAGAGGAAGAGGAAGAGGACGTGGCCGTGGCAGAGGAAG  457

Query  564   aggGGGTCCTAGGCGATAATGTCTCTCAAGATTTCAAAGTCATATGAGATTTGGGATATT  623
             ||||||||||||||||||||||||||||||||||||||||||||||||||||||||||||
Sbjct  458   AGGGGGTCCTAGGCGATAATGTCTCTCAAGATTTCAAAGTCATATGAGATTTGGGATATT  517
```

The sequencing chromatogram (A) and BLAST analysis (B) of small nuclear ribonucleoprotein D1 polypeptide (SNRPD1). The cDNA sequence of plasmid DNA isolated from yeast cells selected with two-hybrid assay was applied to BLAST analysis provided by the NCBI.
