# Supplementary material for: Human DDX56 protein interacts with influenza A virus NS1 protein and stimulates the virus replication
Source: Genet Mol Biol. 2021 Mar 22;44(1):e20200158. doi: 10.1590/1678-4685-GMB-2020-0158 (PMC7983190; doi:10.1590/1678-4685-GMB-2020-0158)
Supplement: Figure S6 - [file 1415-4757-GMB-44-1-e20200158-s6.pdf]

Genetics and Molecular Biology

https://doi.org/10.1590/1678-4685-GMB-2020-0158

"Supplementary Material to "Human DDX56 Protein Interacts with Influenza A Virus NS1 Protein and Stimulates the Virus Replication"

Figure S6 - The top five I-TASSER 3D models of human DDX56 protein.

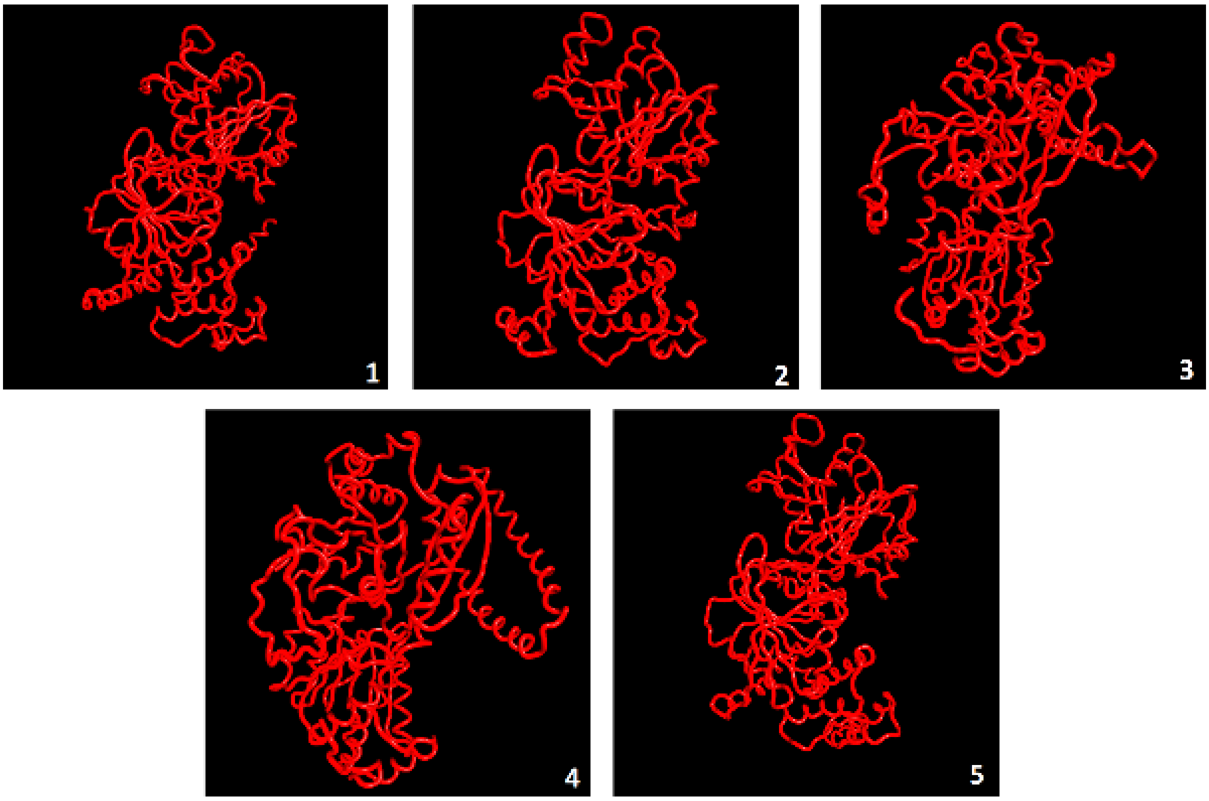

The top five I-TASSER 3D models of human DDX56 protein. The C-score of the models: -1.05 (model 1), -1.44 (model 2), -2.27 (model 3), -3.22 (model 4), -2.31 (model 5).
