# Supplementary material for: Human DDX56 protein interacts with influenza A virus NS1 protein and stimulates the virus replication
Source: Genet Mol Biol. 2021 Mar 22;44(1):e20200158. doi: 10.1590/1678-4685-GMB-2020-0158 (PMC7983190; doi:10.1590/1678-4685-GMB-2020-0158)
Supplement: Figure S7 - [file 1415-4757-GMB-44-1-e20200158-s7.pdf]

Genetics and Molecular Biology

https://doi.org/10.1590/1678-4685-GMB-2020-0158

"Supplementary Material to "Human DDX56 Protein Interacts with Influenza A Virus NS1 Protein and Stimulates the Virus Replication"

Figure S7 - The top five I-TASSER 3D models of influenza A virus NS1 protein.

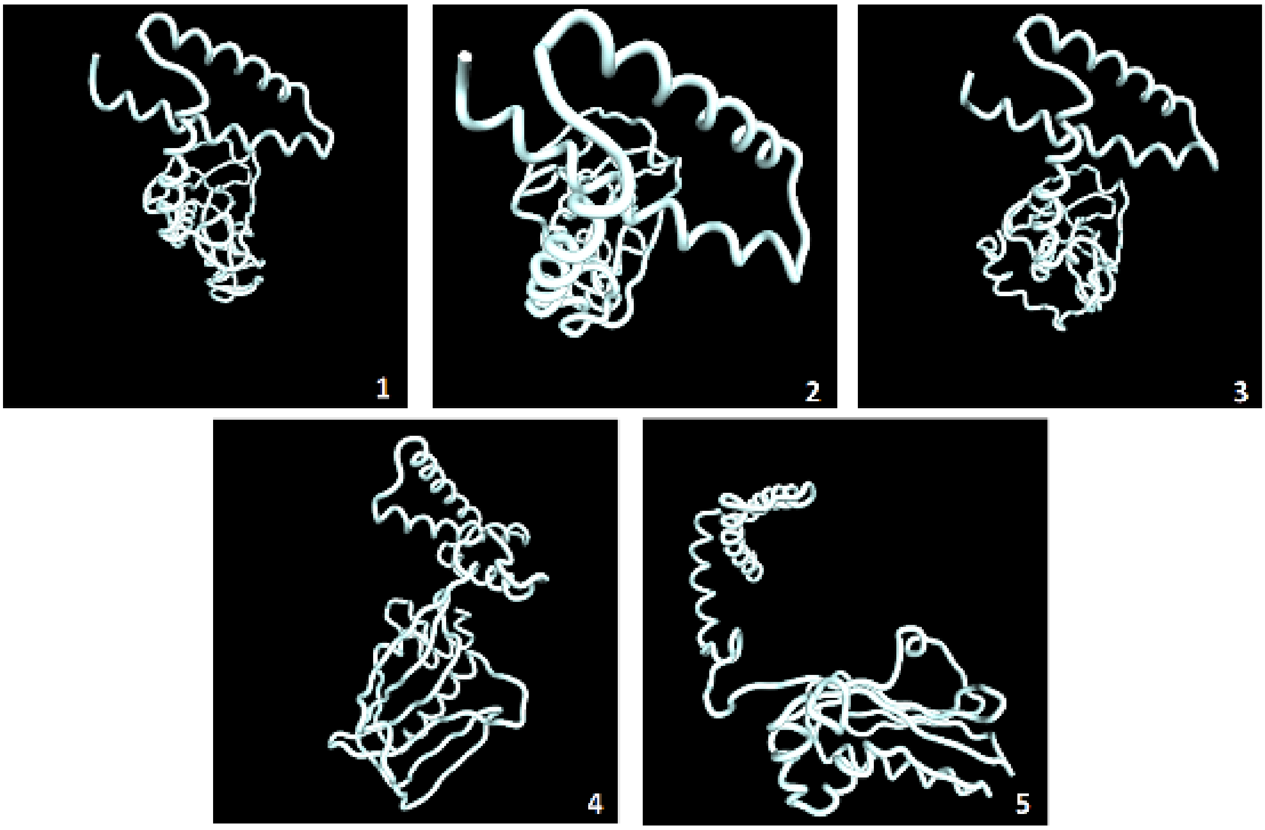

The top five I-TASSER 3D models of influenza A virus NS1 protein. The C-score of the models: -0.59 (model 1), -1.17 (model 2), -1.36 (model 3), -2.99 (model 4), -0.80 (model 5).
