# Supplementary material for: Human DDX56 protein interacts with influenza A virus NS1 protein and stimulates the virus replication
Source: Genet Mol Biol. 2021 Mar 22;44(1):e20200158. doi: 10.1590/1678-4685-GMB-2020-0158 (PMC7983190; doi:10.1590/1678-4685-GMB-2020-0158)
Supplement: Figure S3 - [file 1415-4757-GMB-44-1-e20200158-s3.pdf]

Genetics and Molecular Biology

https://doi.org/10.1590/1678-4685-GMB-2020-0158

"Supplementary Material to "Human DDX56 Protein Interacts with Influenza A Virus NS1 Protein and Stimulates the Virus Replication"

Figure S3 - The sequencing chromatogram (A) and BLAST analysis (B) of proteasome subunit beta type 4 (PSMB4).

A

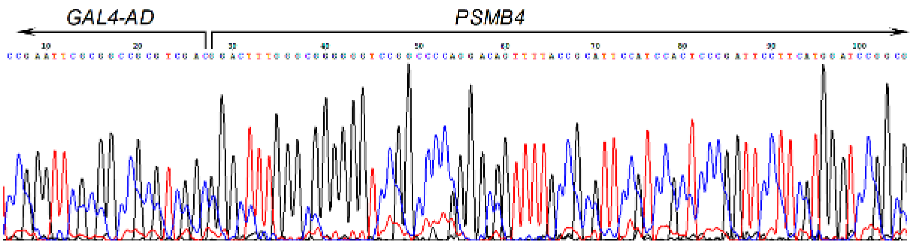

B

31.10.2020 NCBI Blast:Nucleotide Sequence

Homo sapiens proteasome 20S subunit beta 4 (PSMB4), mRNA
Sequence ID: **NM_002796.3** Length: 918 Number of Matches: 1
Range 1: 42 to 755

Score	Expect	Identities	Gaps	Strand	Frame
1308 bits(708)	0.0()	712/714(99%)	0/714(0%)	Plus/Plus	

```
Query  27   CGGACTTTGGGCGGGGGTCCGGCCCCAGGACAGTTTTACCGCATTCCATCCACTCCCGA  86
            ||||||||||||||||||||||||||||||||||||||||||||||| ||||||||||||
Sbjct  42   CGGACTTTGGGCGGGGGTCCGGCCCCAGGACAGTTTTACCGCATTCCGTCCACTCCCGA  101

Query  87   TTCCTTCATGGATCCGGCGTCTGCACTTTACAGAGGTCCAATCACGCGGACCCAGAACCC  146
            ||||||||||||||||||||||||||||||||||||||||||||||||||||||||||||
Sbjct  102  TTCCTTCATGGATCCGGCGTCTGCACTTTACAGAGGTCCAATCACGCGGACCCAGAACCC  161

Query  147  CATGGTGACCGGGACCTCAGTCCTCGGCGTTAAGTTCGAGGGCGGAGTGGTGATTGCCGC  206
            ||||||||||||||||||||||||||||||||||||||||||||||||||||||||||||
Sbjct  162  CATGGTGACCGGGACCTCAGTCCTCGGCGTTAAGTTCGAGGGCGGAGTGGTGATTGCCGC  221

Query  207  AGACATGCTGGGATCCTACGGCTCCTTGGCTCGTTTCCGCAACATCTCTCGCATTATGCG  266
            ||||||||||||||||||||||||||||||||||||||||||||||||||||||||||||
Sbjct  222  AGACATGCTGGGATCCTACGGCTCCTTGGCTCGTTTCCGCAACATCTCTCGCATTATGCG  281

Query  267  AGTCAACAACAGTACCATGCTGGGTGCCTCTGGCGACTACGCTGATTTCCAGTATTTGAA  326
            ||||||||||||||||||||||||||||||||||||||||||||||||||||||||||||
Sbjct  282  AGTCAACAACAGTACCATGCTGGGTGCCTCTGGCGACTACGCTGATTTCCAGTATTTGAA  341

Query  327  GCAAGTTCTCGGCCAGATGGTGATTGATGAGGAGCTTCTGGGAGATGGACACCGCTATAG  386
            |||||||||||||||||||||||||||||||||||||||||||||||||||| |||||||
Sbjct  342  GCAAGTTCTCGGCCAGATGGTGATTGATGAGGAGCTTCTGGGAGATGGACACAGCTATAG  401

Query  387  TCCTAGAGCTATTCATTCATGGCTGACCAGGGCCATGTACAGCCGGCGCTCGAAGATGAA  446
            ||||||||||||||||||||||||||||||||||||||||||||||||||||||||||||
Sbjct  402  TCCTAGAGCTATTCATTCATGGCTGACCAGGGCCATGTACAGCCGGCGCTCGAAGATGAA  461

Query  447  CCCTTTGTGGAACACCATGGTCATCGGAGGCTATGCTGATGGAGAGAGCTTCCTCGGTTA  506
            ||||||||||||||||||||||||||||||||||||||||||||||||||||||||||||
Sbjct  462  CCCTTTGTGGAACACCATGGTCATCGGAGGCTATGCTGATGGAGAGAGCTTCCTCGGTTA  521
```

The sequencing chromatogram (A) and BLAST analysis (B) of proteasome subunit beta type 4 (PSMB4). The cDNA sequence of plasmid DNA isolated from yeast cells selected with two-hybrid assay was applied to BLAST analysis provided by the NCBI.
